# Headspace with Gas Chromatography-Mass Spectrometry for the Use of Volatile Organic Compound Profile in Botanical Origin Authentication of Honey

**DOI:** 10.3390/molecules28114297

**Published:** 2023-05-24

**Authors:** Ana Castell, Natalia Arroyo-Manzanares, Yolanda Guerrero-Núñez, Natalia Campillo, Pilar Viñas

**Affiliations:** Department of Analytical Chemistry, Faculty of Chemistry, Regional Campus of International Excellence “Campus Mare Nostrum”, University of Murcia, E-30100 Murcia, Spain; ana.castell@um.es (A.C.); yolanda.guerreron@um.es (Y.G.-N.); ncampi@um.es (N.C.)

**Keywords:** honey authenticity, botanical origin, volatile compounds, orange blossom, albaida, thousand flower, rosemary, HS-GC-MS, chemometrics

## Abstract

The botanical origin of honey determines its composition and hence properties and product quality. As a highly valued food product worldwide, assurance of the authenticity of honey is required to prevent potential fraud. In this work, the characterisation of Spanish honeys from 11 different botanical origins was carried out by headspace gas chromatography coupled with mass spectrometry (HS-GC-MS). A total of 27 volatile compounds were monitored, including aldehydes, alcohols, ketones, carboxylic acids, esters and monoterpenes. Samples were grouped into five categories of botanical origins: rosemary, orange blossom, albaida, thousand flower and “others” (the remaining origins studied, due to the limitation of samples available). Method validation was performed based on linearity and limits of detection and quantification, allowing the quantification of 21 compounds in the different honeys studied. Furthermore, an orthogonal partial least squares-discriminant analysis (OPLS-DA) chemometric model allowed the classification of honey into the five established categories, achieving a 100% and 91.67% classification and validation success rate, respectively. The application of the proposed methodology was tested by analysing 16 honey samples of unknown floral origin, classifying 4 as orange blossom, 4 as thousand flower and 8 as belonging to other botanical origins.

## 1. Introduction

Honey is a natural substance recognised for its many health benefits. It is considered a complex solution since its composition depends on several factors, including geographical and botanical origin and production process. Consequently, the beneficial effects are also dependent on phytochemical composition [1]. Depending on the botanical origin, three types of honey can be distinguished: monofloral and multifloral honey (from plant nectar) and honeydew (from plant secretions).

The origin of honey is closely related to market price and product quality. Generally, monofloral honey (from the nectar of a single plant species) has a more defined taste and aroma than multifloral honey (from the nectar of several plant species), which results in a higher price. As a high-value food product, honey is often subject to fraud, including adulteration and mislabelling of its origin [2]. The authenticity of honey in terms of botanical origin was traditionally determined by melissopalynological analysis based on pollen structure. However, this technique is difficult to apply for routine analysis as it requires much time and trained personnel [3]. At present, high-performance liquid chromatography (HPLC) [4,5,6], gas chromatography (GC) [7,8,9] and the analysis of physicochemical parameters such as colour, electrical conductivity, moisture, pH or the content of hydroxymethylfurfural (HMF) [7,8,10,11] are the most commonly used techniques for this purpose [12]. Other techniques applied to botanical origin characterisation involve fluorescence spectroscopy [13], near-infrared (NIR) and mid-infrared (MIR) [14] spectroscopy, electronic nose (EN) [14], attenuated total reflectance–Fourier transform infrared (ATR-FTIR) [15], inductively coupled plasma mass spectrometry (ICP-MS) [16,17] and inductively coupled plasma optical emission spectroscopy (ICP-OES) [18,19].

Volatile organic compounds (VOC) analysis to assess the botanical origin of honey has become an effective approach, as the presence and amount of these compounds in honey are closely related to the plant of origin [12]. To date, more than 600 volatile and semi-volatile compounds have been identified in honey [20] from several chemical families including aldehydes, alcohols, ethers, carboxylic acids, ketones, terpenes, phenolic compounds, norisoprenoids and pyran and furan derivatives. Some of these VOCs are considered "floral markers" because they are characteristic of a particular floral origin and are therefore only present in certain types of honey [21,22].

The volatile profile of honey has most frequently been determined using GC coupled with mass spectrometry (MS). Other detectors such as olfactometry [23] and ion mobility spectrometry (IMS) [24,25] have also been used to provide the characteristic fingerprint for botanical origin determination. Regarding the isolation of honey VOCs, different procedures such as Likens–Nickerson steam distillation–extraction [26], micro-simultaneous steam distillation–solvent extraction [27] or ultrasound-assisted extraction (USE) [28] were traditionally carried out. However, these techniques involve the use of solvents and high temperatures, which can lead to the Maillard reaction modifying the volatile composition of honey due to the formation of pyran and furan derivatives [29]. Headspace solid-phase microextraction (HS-SPME) has been widely applied for this purpose [7,9,30]. Despite the advantages provided by this technique, its efficiency can be influenced by different parameters related to analyte absorption and desorption such as time, temperature, agitation, fibre coating, matrix modification or the amount of sample [31].

The aim of this work is the characterisation of Spanish honeys including monofloral and multifloral varieties from 11 different botanical origins (thousand flower, orange blossom, albaida, heather, thyme, orange blossom–lemon, Spanish lavender, melon, broom, oak and rosemary). The total profile of the samples was studied for VOC determination and for classification of honey according to botanical origin using chemometric tools. The novelty of this work lies in the study and classification of a wide variety of honeys by a simple HS-GC-MS analysis, avoiding the use of solvents and tedious procedures, as no sample treatment is required. This work is presented as an efficient methodology for botanical classification of honey in order to avoid honey fraud and is also intended to increase knowledge of honey composition and contribute to the identification of possible floral markers.

## 2. Results and Discussion

### 2.1. HS-GC-MS Method Optimisation

The first step was a comprehensive optimisation of the proposed analytical method to achieve efficient VOC extraction from the investigated Spanish honeys. The optimised parameters were oven programme, injection mode and volume, incubation time and temperature, amount of honey and the addition of NaCl to the sample. Optimisation was carried out using an orange blossom honey sample as a reference matrix. First, experiments were carried out using 1 g of honey fortified at 1 μg g^−1^ of the 37 VOC standards investigated (specified in Section 3.1). Then, honey was incubated at 100 °C and 750 rpm for 10 min, and 1 mL of headspace was injected into the system in splitless mode. The volatile profile of honey was used to study the response of the different conditions.

Firstly, the oven programme was investigated to achieve an adequate peak separation in a shorter elution time. As no peak was observed at times over 35 min, the oven programme finally used was as follows: 40 °C (5 min), increased to 130 °C at 5 °C min^−1^ and then 200 °C at 35 °C min^−1^.

Subsequently, the sample injection mode was optimised by performing splitless and split experiments at ratios of 10:1, 20:1, 50:1 and 100:1. The highest intensities and peak resolutions were obtained from the lowest dilution ratio. Therefore, the split ratio was set at 10:1. Regarding the injection volume, three different volumes were tested: 1, 1.5 and 2 mL. As was expected, signal intensity increased with injection volume; however, no significant differences were found between 1.5 and 2 mL, and thus the volume selected was 1.5 mL.

The amount of honey studied ranged from 0.5–5 g. As the amount of honey increased, the number and intensity of signals increased (Figure S1). However, 3 g of honey was finally selected as optimal in order to avoid possible contamination by using higher quantities of sample. The addition of NaCl to honey was investigated in a range of 0–10% to improve VOC extraction by increasing the ionic strength of the medium. As a result, the presence of NaCl resulted in lower peak areas and was therefore rejected for honey analysis.

The next parameters were the incubation time and temperature of honey samples. Firstly, the time of incubation was studied in the 5–20 min range, achieving higher peak intensities at longer incubation times; thus, 20 min was selected as the optimal time. Finally, incubation temperature ranged from 80 to 110 °C. No significant differences were obtained from 90 °C onwards, and this temperature was therefore established as optimal for honey incubation. Higher temperatures were not tested to avoid forming derivative compounds which modify the volatile profile of honey.

### 2.2. Monitorisation of VOCs in Honey

The proposed HS-GC-MS method was used to analyse 31 honey samples from the 11 different botanical origins investigated. For the characterisation of the different honeys, the identification of their VOCs was carried out. For this purpose, 37 standards of VOCs previously identified in honey were analysed [31,32], including seven alcohols (1-octanol, 1-hexanol, 1-pentanol, 3-methyl-1-butanol, 1-penten-3-ol, trans-2-hexen-1-ol and 2-octanol), two carboxylic acids (butyric acid and dodecanoic acid), fourteen aldehydes (3,4,5-trimethoxybenzaldehyde, 3,4-dimethoxybenzaldehyde, trans-2-heptenal, benzaldehyde, hexanal, trans-2-octenal, heptanal, nonanal, octanal, p-anisaldehyde, trans-2-decenal, trans-2-hexen-1-al, valeraldehyde and trans-2-pentenal), nine ketones (6-methyl-5-hepten-2-one, 2-pentanone, 2-butanone, 2-heptanone, 2-hexanone, 2-nonanone, 2-octanone, 4-methylacetophenone and 4-methylpentan-2-one), three esters (ethyl isovalerate, ethyl butyrate and ethyl acetate) and two monoterpenes (limonene and linalool). In addition, toluene and p-xylene were used as internal standards (IS).

As a result, the proposed methodology allowed the monitoring of 27 VOCs. Table 1 summarises the retention time and the target and qualifiers ions of these compounds. 

**Figure 1:**
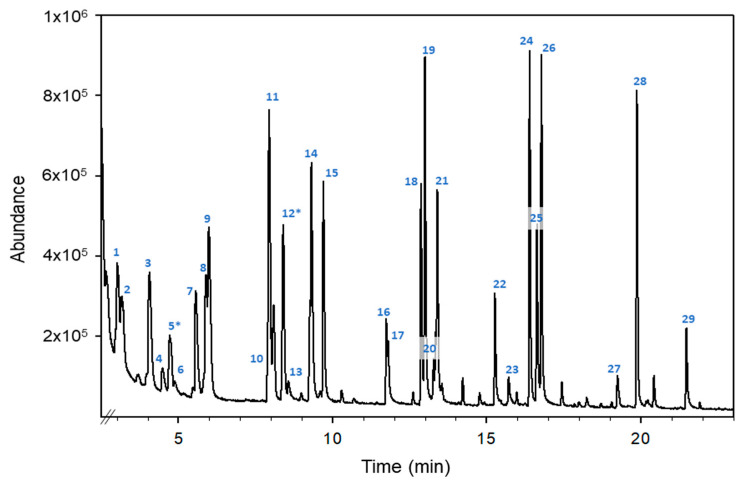
HS-GC-MS total ion chromatogram of an orange blossom honey sample fortified with a mixture of monitored VOCs at 1 µg g^−1^. Compounds: (1) 2-pentanone, (2) valeraldehyde, (3) 4-methyl-pentan-2-one, (4) trans-2-pentenal, (5*) toluene, (6) 1-pentanol, (7) 2-hexanone, (8) hexanal, (9) ethyl butyrate, (10) trans-2-hexen-1-al, (11) ethyl isovalerate, (12*) p-xylene, (13) 1-hexanol, (14) 2-heptanone, (15) heptanal, (16) trans-2-heptenal, (17) benzaldehyde, (18) 6-methyl-5-hepten-2-one, (19) 2-octanone, (20) 2-octanol, (21) octanal, (22) trans-2-octenal, (23) 1-octanol, (24) 2-nonanone, (25) linalool, (26) nonanal, (27) 4-methylacetophenone, (28) decanal and (29) trans-2-decenal. The superscript “*” means the compounds used as internal standards.

Figure 1 shows the total ion chromatogram (TIC) obtained for an orange blossom honey sample fortified at 1 μg g^−1^ with the mixture using the monitored standards.

### 2.3. Method Characterisation and Quantification of Identified VOCs

The determination of monitored compounds in honey samples was carried out by the construction of calibration curves using refined oil fortified at eight concentration levels ranging from 0.02 to 1 μg g^−1^ in duplicate. Samples were also fortified with toluene and p-xylene as IS at a constant concentration of 0.1 μg g^−1^.

Calibration curves were constructed by plotting each compound peak area ratio in terms of IS peak area versus compound concentration. Due to the retention times of volatile compounds, toluene (RT = 4.70 min) was the selected IS for compounds eluting before 8 min (2-pentanone, valeraldehyde, 4-methylpentan-2-one, ethyl isovalerate, 2-hexanone, trans-2-hexen-1-al, hexanal, trans-2-pentenal, ethyl butyrate and 1-pentanol); and p-xylene (RT = 8.39 min) for compounds with retention times from 8 min onwards (1-hexanol, 2-octanol, 2-heptanone, 6-methyl-5-hepten-2-one, heptanal, octanal, trans-2-heptenal, 1-octanol, benzaldehyde, 2-octanone, trans-2-octenal, 2-nonanone, nonanal, 4-methylacetophenone, trans-2-decenal, decanal and linalool). Furthermore, limits of detection (LODs) and quantification (LOQs) were calculated for signal/noise ratios of 3 and 10, respectively. Table 2 contains linear ranges and values of LOD and LOQ for each compound. Good linearities were achieved for all the monitored compounds, being in all cases R2 > 0.98, and LOQ values ranging from 0.015 to 0.249 μg g^−1^.

The optimised and validated method was applied for VOC quantification in honey samples from different botanical origins. All samples were analysed in duplicate. Table 3 shows the mean content, the range of concentrations and the percentage of occurrence of each compound in the different honeys. The mean and occurrence were calculated considering only concentrations above the corresponding LOQs. Although thirteen different honeys were available, five groups were established according to botanical origin, due to the limited number of samples from certain origins which were included in the same group. Thus, honey was classified as albaida, orange blossom (including orange blossom-lemon), thousand flower, rosemary or honey from other origins (including Spanish lavender, heather, melon, broom, oak and thyme).

To determine which compounds are relevant to each category of honey, one-way analysis of variance (ANOVA) and least significant difference (LSD) tests were conducted. Based on the results, ethyl butyrate and 2-heptanone are volatile compound characteristic of albaida and orange blossom honey, respectively, since these compounds were not detected in any other type of honey. Linalool allows the differentiation of orange blossom honey from the other honeys as it is classified in a separate group according to the LSD test, with an average concentration of 35 ± 19 ng g^−1^. Similarly, decanal showed the highest content in rosemary honey (260 ± 446 ng g^−1^). On the other hand, trans-2-heptenal and trans-2-pentanal are only detected in the category “other origins” and, therefore, albaida, orange blossom, rosemary and thousand flower honeys do not contain them. Similarly, albaida, orange blossom and rosemary honeys do not contain 4-methylpentan-2-one; 2-nonanone was not found in orange blossom, thousand flower and rosemary. The compound 2-hexanone was detected below the LOQ in all cases. 2-Octanone showed the highest quantity in rosemary honey (56.2 ± 0.3 ng g^−1^) and the lowest in thousand flower honey (20.5 ± 0.4 ng g^−1^). Finally, no significant differences were found for octanal, nonanal and 4-methylacetophenone among the different honey categories. The compounds 2-pentanone, 6-methyl-5-hepten-2-one, valeraldehyde, 2-hexanone, octanal, hexanal, nonanal, benzaldehyde and linalool were also previously detected in honeys from the same origins by HS-GC-IMS [25].

Due to the variability of the compound contents found within a group, the assignment of marker compounds for a specific honey was not feasible. Thus, chemometric techniques were investigated for honey classification according to botanical origin.

### 2.4. Non-Targeted Approach Using GC-MS Data

A non-targeted analysis of honeys was carried out using GC-MS data in order to investigate other features of honey samples besides the identified and quantified VOCs. For this purpose, peak detection, deconvolution and alignment treatments were applied to the data. First, peak detection was performed using 1000 a.u. of amplitude as the minimum peak height, and a mass slice width and mass accuracy for centroiding of 0.5 Da. Data were smoothed using the “linear weighted moving average” approach with 3 scans of smoothing level and 20 scans of an average peak width. Peak deconvolution was carried out by setting a sigma window of 0.5 to obtain the resolved chromatographic peaks, avoiding the detection of noise. Finally, peak alignment was performed based on the RT with a tolerance of 0.075 min and a 70% similarity threshold. As a result, 274 features were detected: the 27 monitored VOCs of honey, the 2 IS compounds (toluene and p-xylene) and 245 non-identified compounds. The detected peaks of identified and non-identified compounds were used for chemometric analysis.

### 2.5. Chemometric Model for the Classification of Honey According to Botanical Origin

In order to investigate honey sample classification according to the botanical origin, an orthogonal partial least squares–discriminant analysis (OPLS-DA) using the identified and non-identified compounds was carried out. For this purpose, the 31 available samples analysed in duplicate were used to classify them into the 5 established groups of floral origins: albaida, orange blossom, thousand flower, rosemary and others. Thus, the data matrix was composed of 62 sample analyses (rows) × 274 features of MS (columns).

**Figure 2:**
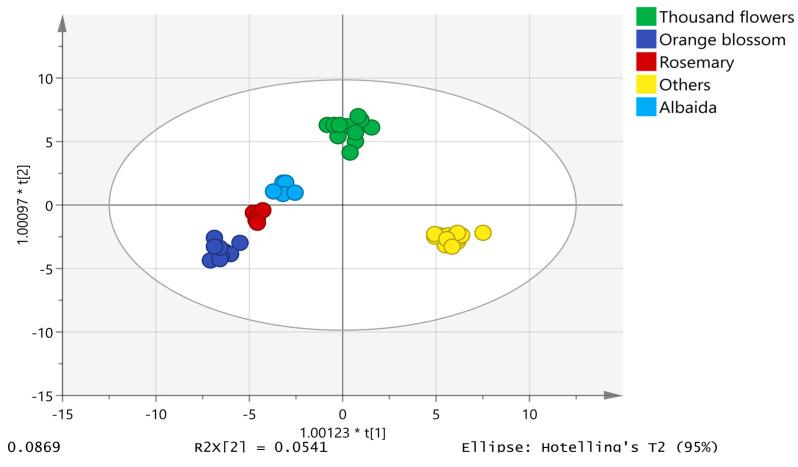
OPLS-DA model for honey discrimination according to botanical origin.

The chemometric model was built using the unit variance (UV) scale, also known as “autoscaling”. The honey samples displayed a normal distribution of data in a normal probability plot of residues. Eighty percent of the data was used for model training consisting of 50 analyses of albaida (5), orange blossom (8), thousand flower (13), rosemary (5) and others (19). The other 20% of the data was applied to validate the model; therefore, the validation set included 12 analyses (1 from albaida, 2 from orange blossom, 3 from thousand flower, 1 from rosemary and 5 from other origins). The proposed model was composed of 4 + 13 + 0 components with an R2X = 0.850, R2Y = 0.967 and a model prediction index (Q2) of 0.641, demonstrating the good predictive ability of the model. Figure 2 shows the two-dimensional scatter plot of the first component against the second component, which explains the largest variation of the X space. Honey samples were successfully classified into the five established botanical origin categories, achieving a 100% and 91.67% classification and validation success rate, respectively. Only the rosemary chromatogram was misclassified in the category of thousand flower in the validation set (Table S1).

An analysis of variable importance in projection (VIP) was conducted to identify the compounds which most influence the classification of honey into the five established categories. As VIP values greater than 1 are considered influential values, 121 features including hexanal, 2-heptanone, 4-methylacetophenone, 1-hexanol, 1-octanol, ethyl isovalerate, 2-hexanone, trans-2-octenal, 6-methyl-5-hepten-2-one, 2-nonanone, 2-octanol, 4-methylpentan-2-one and linalool were deemed key compounds. A loadings scatter plot of the model was also performed to evaluate the relationship between the Y variables and X variables of the predictive components. Higher loading values lead to higher contributions to model building. Figure S2 shows which categories of honey provide similar information to the model and the relationship of each one to the investigated MS features. As can be seen in Figure S2, “thousand flower” is the most influential category. Thousand flower, orange blossom and other honeys differ significantly more than albaida and rosemary, which provide similar information to the model. Regarding the MS features, the two most influential markers for each honey are also indicated in Figure S2. In the case of thousand flower honey, the markers were the number “274” (RT = 24.72 min; quant mass (QM) = 161 *m/z*) and “181” (RT = 12.37 min; QM=93 *m/z*); for albaida honey, the markers were “183” (RT = 12.63 min; QM = 71 *m/z*) and “261” (RT = 21.92 min; QM = 71 *m/z*); the numbers “252” (RT = 20.29 min; QM = 91 *m/z*) and “237” (RT = 18.96 min; QM = 94 *m/z*) were for rosemary honey; “157” (RT = 9.36 min; QM = 95 *m/z*) and 2-heptanone were for orange blossom; and “42” (RT = 2.14 min; QM = 82 *m/z*) and “45” (RT = 2.18 min; QM = 82 *m/z*) were for other honey origins.

### 2.6. Application of the Proposed Method

Method applicability was validated by the analysis of 16 samples of honey of unknown floral origin. Honey samples were analysed using the proposed HS-GC-MS method in duplicate, resulting in 32 analyses in total. Then, the chromatograms were introduced into the proposed OPLS-DA model for their classification based on honey characterisation. Consequently, 4 samples were classified as thousand flower honey, 4 as orange blossom honey and 8 as belonging to other floral origins.

## 3. Materials and Methods

### 3.1. Standards and Solvents

The 37 standards monitored for VOC identification in honey were provided by Sigma-Aldrich (St. Louis, MO, USA): seven alcohols (1-octanol, 1-hexanol, 1-pentanol, 3-methyl-1-butanol, 1-penten-3-ol, trans-2-hexen-1-ol and 2-octanol), two carboxylic acids (butyric and dodecanoic acid), fourteen aldehydes (3,4,5-trimethoxybenzaldehyde, trans-2-hexen-1-al, 3,4-dimethoxybenzaldehyde, trans-2-octenal, benzaldehyde, trans-2-heptenal, hexanal, heptanal, nonanal, octanal, p-anisaldehyde, trans-2-decenal, trans-2-pentenal and valeraldehyde), nine ketones (2-hexanone, 2-butanone, 4-methylpentan-2-one, 2-heptanone, 2-nonanone, 2-pentanone, 2-octanone, 4-methylacetophenone and 6-methyl-5-hepten-2-one), three esters (ethyl isovalerate, ethyl butyrate and ethyl acetate) and two monoterpenes (limonene and linalool). Individual solutions of each standard were prepared in methanol at 1000 mg L^−1^ and stored at 4 °C. The compounds used as internal standards (toluene and p-xylene) were also supplied by Sigma-Aldrich.

Methanol was supplied by ThermoFisher Scientific (MA, USA), and sodium chloride (NaCl) was purchased from Sigma-Aldrich. Helium from Messer (Madrid, Spain) was used as a carrier gas.

### 3.2. Honey Samples

The different varieties of monofloral and multifloral honey were provided by several beekeepers from Murcia (Spain). Specifically, 31 honey samples from 11 botanical origins were analysed, namely orange blossom (five samples), albaida (three samples), heather (two samples), orange blossom–lemon (one sample), Spanish lavender (one sample), melon (two samples), thousand flower (eight samples), broom (two sample), oak (two sample), rosemary (three samples) and thyme (two samples) honeys. Furthermore, 16 samples of unknown origin were purchased from local markets and analysed to evaluate the applicability of the proposed method. All honeys were kept in the dark at 4 °C until analysis.

### 3.3. Instrumentation and Software

An 8890-gas chromatograph from Agilent Technologies (CA, USA) with a multi-purpose sampler (MPS) operating in headspace mode and a 2.5 mL syringe (Gerstel, Mülheim, Germany) were coupled to a 5977B-quadrupole mass spectrometer with an inert ion source also from Agilent. Chromatographic separation was performed using two in-line Agilent HP-5MS capillary columns (5% diphenyl-95% dimethylpolysiloxane) with 15 m × 0.25 mm I.D. × 0.25 μm, combined with a backflush system setting 0.83 min as post run time.

MassHunter Workstation software (Qualitative Analysis version B.08.00) from Agilent Technologies was used for data acquisition. StatGraphics Plus 5.1 (Statistical Graphics, Rockville, MD, USA), MS-DIAL 4.80 and SIMCA 14.1 (Umetrics, Umeå, Sweden) software were used for the processing of data. The NIST mass spectral library was used for the identification of VOCs.

For the homogenization of honey samples before analysis, an LLG-uniTEXER vortex agitator (Heathrow Scientific, Vernon Hills, Chicago, IL, USA) was used.

### 3.4. HS-GC-MS Analysis

Honey was tempered at room temperature before analysis and 3 g was weighed into a 20 mL vial. Then, 100 μL of MeOH and 30 μL of the IS solution at 10 mg L^−1^ were added. This mixture was shaken for 1 min by vortex at 1500–2000 rpm for homogenisation. Subsequently, the vial containing the sample was incubated at 90 °C for 20 min at 750 rpm and 1.5 mL of the headspace was injected into the GC system. The injection temperature was set to 100 °C at a ratio of 10:1 (split mode). The flow of the carrier gas, helium, was 1 mL min^−1^. The GC oven programme started at 40 °C (5 min), increased to 130 °C at 5 °C min^−1^ and then 200 °C at 35 °C min^−1^, resulting in a total runtime of 25 min. The ion source, transfer line and quadrupole temperatures were 230, 300 and 150 °C, respectively.

The MS was performed in electron impact (EI) mode (70 eV) and experiments were carried out using scan mode in a range of 35–500 *m/z*. The quantification of compounds was performed using selected ion monitoring (SIM) mode and the extract ion chromatograms (EIC) of the target ions.

### 3.5. Data Processing

First, HS-GC-MS data were converted to Analysis Base Framework (ABF) format for data processing using MS-DIAL. The pre-treatment of the total ion chromatograms (TICs) involved peak detection, deconvolution and alignment treatment of data. The detected peaks of identified and non-identified compounds were used for chemometric analysis in order to differentiate honey according to botanical origin. The chemometric model based on orthogonal partial least squares–discriminant analysis (OPLS-DA) was constructed with SIMCA software using the unit variance (UV) scale. Model building was carried out using a training dataset consisting of 80% of data, selected randomly, and validation was performed with a validation dataset using the remaining 20%. The parameters of R2X (cum), R2Y (cum) and Q2 (cum) were evaluated to assess the adequacy of the model. R2X and R2Y correspond to the cumulative fraction of variance in X and Y explained by a particular component, and Q2 (cum) represents the predictive ability of the model. The range of these parameters is 0 to 1 [33]. The chemometric model is acceptable at a Q2 value of 0.5 [34]. Sensitivity of the model was defined as ∑ True positive/(∑ True positive + ∑ False negative) × 100.

## 4. Conclusions

The proposed analytical method allowed the characterisation of honey from different botanical origins in a rapid and efficient way without the requirement of sample pre-treatment, which involves a longer process time and additional costs for instruments and reagents. Characterisation was carried out by monitoring 27 volatile compounds, obtaining the average content, concentration range and incidence of these compounds in each honey. This allowed the identification of possible floral markers such as ethyl butyrate for albaida honey and 2-heptanone for orange blossom honey. In addition, the proposed OPLS-DA chemometric model based on a non-targeted analysis of honeys allowed a successful classification according to botanical origin, achieving a classification success rate of 100% and a validation success of 91.67%, demonstrating the suitability of the model for the identification of honeys of unknown origin.

## Figures and Tables

**Table 1:** VOCs monitored in honey using the HS-GC-MS method.

Compound	RT ^1^ (min)	Target Ion (*m/z*)	Qualifier Ions (*m/z*)
2-Pentanone	2.99	43	71, 86
Valeraldehyde	3.16	44	29, 86
4-Methylpentan-2-one	4.03	43	58, 85
Trans-2-pentenal	4.46	55	84, 41
Toluene *	4.70	91	92, 65
1-Pentanol	4.80	42	31, 70
2-Hexanone	5.54	43	58, 85
Hexanal	5.88	44	57, 82
Ethyl butyrate	5.96	71	43, 88
Trans-2-hexen-1-al	7.86	41	69, 83
Ethyl isovalerate	7.93	88	57, 115
p-Xylene *	8.39	91	105, 106
1-Hexanol	8.54	56	43, 84
2-Heptanone	9.32	43	58, 71
Heptanal	9.69	70	44, 96
Trans-2-heptenal	11.73	41	70, 83
Benzaldehyde	11.79	106	77, 107
6-Methyl-5-hepten-2-one	12.86	43	69, 108
2-Octanone	12.99	43	71, 85
2-Octanol	13.33	45	70, 97
Octanal	13.39	41	44, 128
Trans-2-octenal	15.26	41	83, 97
1-Octanol	15.70	56	41, 84
2-Nonanone	16.39	43	71, 99
Linalool	16.63	71	93, 136
Nonanal	16.77	57	43, 98
4-Methylacetophenone	19.25	119	91, 65
Decanal	19.87	43	82, 112
Trans-2-decenal	21.48	43	82, 110

* Compounds used as internal standards. ^1^ RT: retention time.

**Table 2:** Chromatographic parameters of monitored VOCs in honey.

Compound	Linear Range (μg g^−1^)	R^2^	LOD ^1^ (LOQ ^2^) (μg g^−1^)
2-Pentanone	0.016–1.00	0.993	0.005 (0.016)
Valeraldehyde	0.016–1.00	0.992	0.005 (0.016)
4-Methylpentan-2-one	0.016–1.00	0.993	0.005 (0.016)
Trans-2-pentenal	0.216–1.00	0.993	0.065 (0.216)
1-Pentanol	0.249–1.00	0.990	0.075 (0.249)
2-Hexanone	0.218–1.00	0.991	0.065 (0.218)
Hexanal	0.015–1.00	0.991	0.005 (0.015)
Ethyl butyrate	0.040–1.00	0.997	0.012 (0.040)
Trans-2-hexen-1-al	0.129–1.00	0.980	0.039 (0.129)
Ethyl isovalerate	0.040–1.00	0.995	0.012 (0.040)
1-Hexanol	0.083–1.00	0.994	0.025 (0.083)
2-Heptanone	0.083–1.00	0.994	0.025 (0.083)
Heptanal	0.016–1.00	0.991	0.005 (0.016)
Trans-2-heptenal	0.130–1.00	0.999	0.039 (0.130)
Benzaldehyde	0.016–1.00	0.992	0.005 (0.016)
6-Methyl-5-hepten-2-one	0.016–1.00	0.995	0.005 (0.016)
2-Octanone	0.016–1.00	0.996	0.005 (0.016)
2-Octanol	0.083–1.00	0.990	0.025 (0.083)
Octanal	0.016–1.00	0.995	0.005 (0.016)
Trans-2-octenal	0.016–1.00	0.991	0.005 (0.016)
1-Octanol	0.016–1.00	0.991	0.005 (0.016)
2-Nonanone	0.016–1.00	0.997	0.005 (0.016)
Linalool	0.015–1.00	0.994	0.005 (0.015)
Nonanal	0.016–1.00	0.998	0.005 (0.016)
4-Methylacetophenone	0.016–1.00	0.994	0.005 (0.016)
Decanal	0.016–1.00	0.998	0.005 (0.016)
Trans-2-decenal	0.218–1.00	0.995	0.065 (0.218)

^1^ LOD: limit of detection; ^2^ LOQ: limit of quantification.

**Table 3:** Mean concentrations, range and percentage of occurrence of the identified VOCs in honeys.

Compound		Albaida	Orange Blossom	Thousand Flowers	Rosemary	Others
2-Pentanone	Mean (ng g^−1^)	26.0 ± 1.3 ^a^	33 ± 8 ^a,b^	32 ± 9 ^b^	34 ± 7 ^b^	36 ± 10 ^b^
	Range (ng g^−1^)	NQ ^1^–27.4	NQ–45.8	19.4–49.4	26.7–46.0	0.00–61.4
	Incidence (%)	66.7	83.3	100.0	100.0	86.4
Valeraldehyde	Mean (ng g^−1^)	208 ± 38 ^b^	35 ± 15 ^a^	41 ± 24 ^a,b^	46 ± 9 ^a,b^	42 ± 18 ^a^
	Range (ng g^−1^)	NQ–235.2	NQ–69.4	0.00–86.3	34.0–56.9	0.00–69.0
	Incidence (%)	33.3	75.0	94.1	100.0	63.6
4-Methylpentan-2-one	Mean (ng g^−1^)	ND ^2,a^	ND ^a^	46 ± 10 ^b^	ND ^a^	43 ± 8 ^b^
	Range (ng g^−1^)	–	–	0.00–59.1	–	0.00–48.3
	Incidence (%)	–	–	35.3	–	54.5
Trans-2-pentenal	Mean (ng g^−1^)	ND ^a,b^	ND ^a^	ND ^a^	ND ^a,b^	287 ± 38 ^b^
	Range (ng g^−1^)	–	–	–	–	0.00–334.8
	Incidence (%)	–	–	–	–	18.2
2-Hexanone	Mean (ng g^−1^)	NQ	NQ	NQ	NQ	NQ
	Range (ng g^−1^)	NQ	0.00–NQ	0.00–NQ	0.00–NQ	0.00–NQ
	Incidence (%)	–	–	–	–	–
Hexanal	Mean (ng g^−1^)	15.4 ^a^	69 ± 17 ^a^	70 ± 24 ^a^	83 ± 11 ^a^	173 ± 140 ^b^
	Range (ng g^−1^)	0.00–15.4	0.00–94.0	0.00–99.1	74.9–90.5	0.00–463.7
	Incidence (%)	16.7	50.0	47.1	33.3	72.7
Ethyl butyrate	Mean (ng g^−1^)	289 ± 16 ^b^	ND ^a^	ND ^a^	ND ^a^	ND ^a^
	Range (ng g^−1^)	0.00–300.8	–	–	–	–
	Incidence (%)	33.3	–	–	–	–
2-Heptanone	Mean (ng g^−1^)	ND ^a,b^	94 ± 12 ^b^	ND ^a^	ND ^a,b^	ND ^a^
	Range (ng g^−1^)	–	0.00–85.4	–	–	–
	Incidence (%)	–	16.7	–	–	–
Heptanal	Mean (ng g^−1^)	28 ± 3 ^a,b^	27 ± 2 ^a,b^	46 ± 40 ^b^	34 ± 5 ^a,b^	31 ± 5 ^a^
	Range (ng g^−1^)	23.3–32.9	22.2–30.8	0.00–142.1	26.6–39.9	0.00–38.6
	Incidence (%)	100.0	100.0	82.4	100.0	63.6
Trans-2-heptenal	Mean (ng g^−1^)	ND ^a^	ND ^a^	ND ^a^	ND ^a^	84 ± 3 ^a^
	Range (ng g^−1^)	–	–	–	–	0.00–86.8
	Incidence (%)	–	–	–	–	9.1
Benzaldehyde	Mean (ng g^−1^)	NQ ^a^	43 ± 2 ^a^	42 ± 19 ^a,b^	42 ± 11 ^a,b^	102 ± 72 ^b^
	Range (ng g^−1^)	0.00–NQ	0.00–44.4	0.0–80.2	0.00–50.5	0.00–213.9
	Incidence (%)	–	16.7	70.6	66.7	50.0
6-Methyl-5-hepten-2-one	Mean (ng g^−1^)	46.7 ± 0.4 ^a,b^	43 ± 6 ^b^	41.6 ± 0.1 ^a^	41.5 ± 0.3 ^a,b^	48 ± 2 ^a^
	Range (ng g^−1^)	0.00–47.0	0.00–49.1	0.00–41.6	0.00–41.7	0.00–50.1
	Incidence (%)	33.3	66.7	11.8	33.3	18.2
2-Octanone	Mean (ng g^−1^)	25.5 ± 1.4 ^a,b^	20 ± 2 ^a,b^	20.5 ± 0.4 ^a^	56.2 ± 0.3 ^b^	20.8 ± 1.8 ^a,b^
	Range (ng g^−1^)	0.00–26.5	0.00–23.5	0.00–20.8	0.00–56.4	0.00–24.9
	Incidence (%)	33.3	50.0	11.8	33.3	36.4
Octanal	Mean (ng g^−1^)	79 ± 69 ^a^	84 ± 61 ^a^	72 ± 81 ^a^	41 ± 6 ^a^	142 ± 394 ^a^
	Range (ng g^−1^)	31.1–175.7	0.00–161.7	0.00–37.2	0.00–50.6	0.00–1717.8
	Incidence (%)	100.0	75.0	64.7	66.7	81.8
Trans-2-octenal	Mean (ng g^−1^)	34 ± 2 ^a,b^	31 ± 2 ^a,b^	36 ± 3 ^b^	39 ± 9 ^b^	32 ± 5 ^a^
	Range (ng g^−1^)	28.7–36.3	27.0–32.9	0.00–44.3	31.3–55.4	0.00–40.7
	Incidence (%)	100.0	100.0	88.2	100.0	72.7
1-Octanol	Mean (ng g^−1^)	61 ± 2 ^a,b^	89.2 ± 1.8 ^a,b^	84 ± 19 ^a,b^	77 ± 14 ^b^	155 ± 2 ^a^
	Range (ng g^−1^)	0.00–63.2	0.00–90.5	0.00–105.3	0.00–92.0	0.00–156.7
	Incidence (%)	33.3	16.7	23.5	66.7	9.1
2-Nonanone	Mean (ng g^−1^)	38.1 ± 0.2 ^b^	ND ^a^	ND ^a^	ND ^a^	48.3 ± 0.9 ^a,b^
	Range (ng g^−1^)	0.00–38.2	–	–	–	0.00–48.9
	Incidence (%)	33.3	–	–	–	9.1
Linalool	Mean (ng g^−1^)	NQ ^a^	35 ± 19 ^b^	20 ± 2 ^a^	23.8 ± 1.9 ^a^	19.1 ± 1.6 ^a^
	Range (ng g^−1^)	NQ	NQ–72.0	0.00–21.8	NQ–25.1	NQ–21.7
	Incidence (%)	–	66.7	29.4	33.3	27.3
Nonanal	Mean (ng g^−1^)	25.7 ± 1.7 ^a^	29 ± 12 ^a^	72 ± 2 ^a^	30 ± 14 ^a^	97 ± 75 ^a^
	Range (ng g^−1^)	0.00–26.8	0.00–45.9	0.00–96.1	NQ–45.0	0.00–232.4
	Incidence (%)	33.3	50.0	35.3	66.7	36.4
4-Methylacetophenone	Mean (ng g^−1^)	NQ ^a^	57 ± 23 ^a^	70 ± 2 ^a^	82 ± 6 ^a^	161 ± 75 ^a^
	Range (ng g^−1^)	0.00–NQ	0.00–88.1	0.00–72.9	77.2–86.3	0.00–232.4
	Incidence (%)	–	50.0	11.8	33.3	18.2
Decanal	Mean (ng g^−1^)	35.6 ± 0.6 ^a^	36.0 ± 1.0 ^a^	36 ± 2 ^a^	260 ± 446 ^b^	36 ± 2 ^a^
	Range (ng g^−1^)	0.00–36.4	0.00–37.5	0.00–39.1	35.1–929.4	0.00–41.0
	Incidence (%)	66.7	83.3	35.3	66.7	81.8

^1^ NQ means not quantified; ^2^ ND means not detected. The superscripts “^a^” and “^b^” mean the classification into different groups for a specific compound as a result of ANOVA and LSD tests.

## Data Availability

Not applicable.

## Supplementary Materials

The following supporting information can be downloaded at: https://www.mdpi.com/article/10.3390/molecules28114297/s1, Figure S1: Total ion chromatogram of honey during sample amount optimisation by the proposed HS-GC-MS method; Figure S2: Loadings scatter plot of the proposed OPLS-DA model; Table S1: Validation rate of the proposed OPLS-DA model.

## Sample Availability

Sample availability are presented in the manuscript.
