# Supplementary material for: Headspace with Gas Chromatography-Mass Spectrometry for the Use of Volatile Organic Compound Profile in Botanical Origin Authentication of Honey
Source: Molecules. 2023 May 24;28(11):4297. doi: 10.3390/molecules28114297 (PMC10254210; doi:10.3390/molecules28114297)
Supplement: Supplementary file 1 [file molecules-28-04297-s001.zip › molecules-2399163-supplementary.pdf]

Headspace with Gas Chromatography-Mass Spectrometry for the Use of Volatile Organic Compound Profile in Botanical Origin Authentication of Honey

Ana Castell, Natalia Arroyo-Manzanares *, Yolanda Guerrero-Núñez, Natalia Campillo and Pilar Viñas *

Department of Analytical Chemistry, Faculty of Chemistry, Regional Campus of International Excellence "Campus Mare Nostrum", University of Murcia, E-30100 Murcia, Spain

* Correspondence: natalia.arroyo@um.es (N.A.-M.); pilarvi@um.es (P.V.); Tel.:+34-868-887-415 (P.V.)

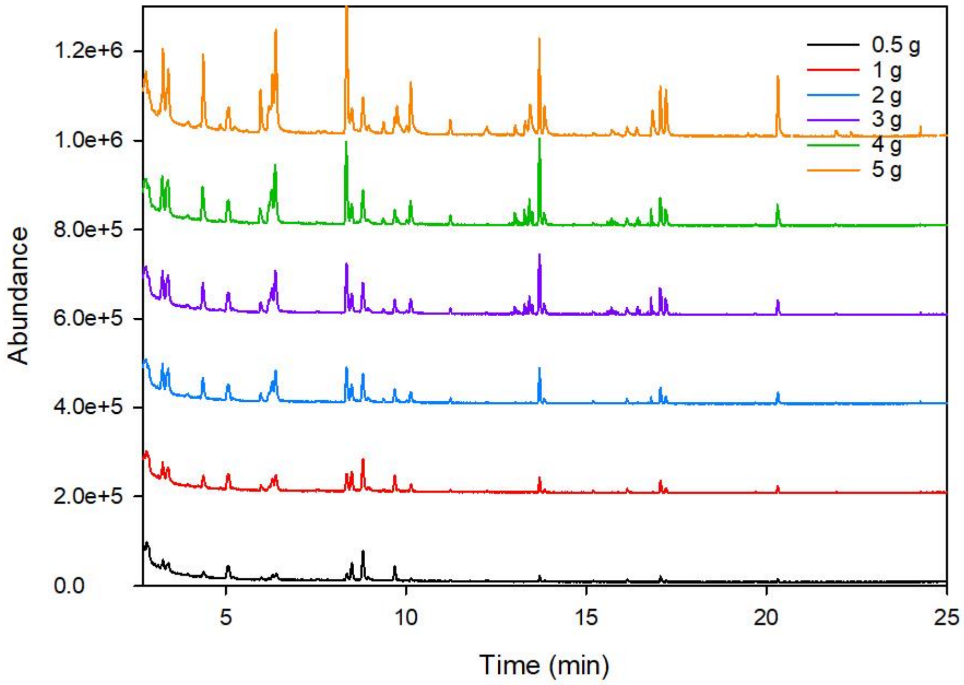

Figure S1. Total ion chromatogram of honey during sample amount optimisation by the proposed HS-GC-MS method.

Table S1. Validation rate of the proposed OPLS-DA model.

Actual/ Prediction	Samples	Orange blossom	Rosemary	Thousand flowers	Albaida	Others	Correct (%)
Orange blossom	2	2	0	0	0	0	100.0
Rosemary	1	0	0	1	0	0	0.0
Thousand flowers	3	0	0	3	0	0	100.0
Albaida	1	0	0	0	1	0	100.0
Others	5	0	0	0	0	5	100.0
Total	12	2	0	4	1	5	91.67

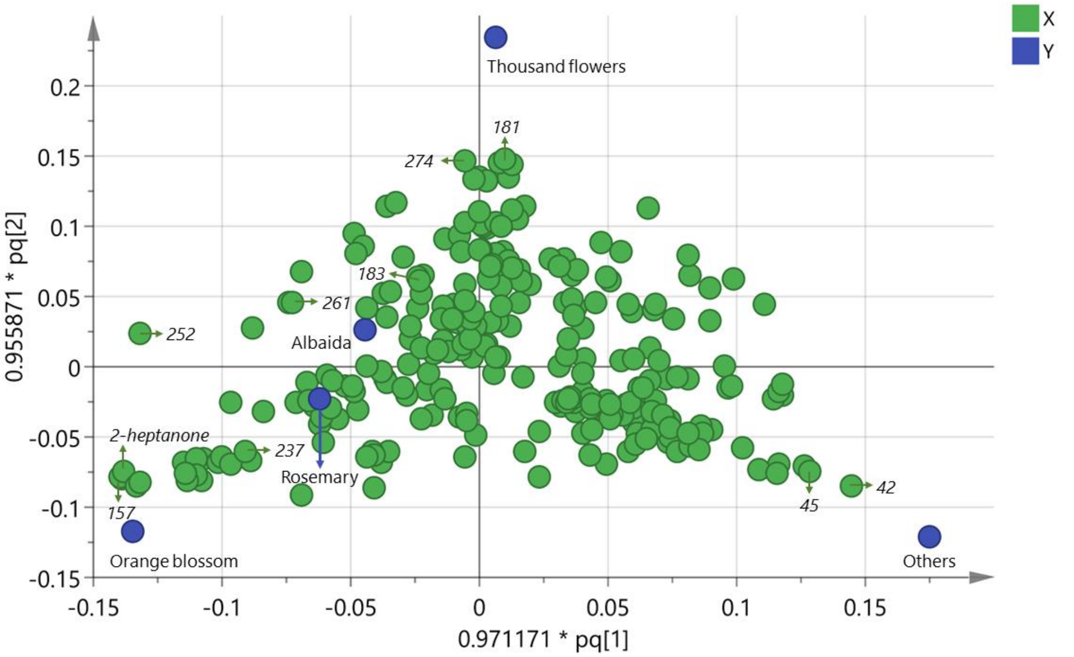

Figure S2. Loadings scatter plot of the proposed OPLS-DA model.
